# Ti_3_C_2_ Nanosheets Functionalized with Ferritin–Biomimetic Platinum Nanoparticles for Electrochemical Biosensors of Nitrite

**DOI:** 10.3390/bios14050258

**Published:** 2024-05-19

**Authors:** Rongqiu Mu, Danzhu Zhu, Gang Wei

**Affiliations:** College of Chemistry and Chemical Engineering, Qingdao University, Qingdao 266071, China; murongqiuname@outlook.com (R.M.); zhudanzhu11@outlook.com (D.Z.)

**Keywords:** Ti_3_C_2_ nanosheets, platinum nanoparticles, apoferritin, NaNO_2_, electrochemical biosensor

## Abstract

Nitrites widely exist in human life and the natural environment, but excessive contents of nitrites will result in adverse effects on the environment and human health; hence, sensitive and stable nitrite detection systems are needed. In this study, we report the synthesis of Ti_3_C_2_ nanosheets functionalized with apoferritin (ApoF)–biomimetic platinum (Pt) nanoparticle (Pt@ApoF/Ti_3_C_2_) composite materials, which were formed by using ApoF as a template and protein-inspired biomineralization. The formed nanohybrid exhibits excellent electrochemical sensing performance towards nitrite (NaNO_2_). Specifically, the Pt@ApoF catalyzes the conversion of nitrites into nitrates, converting the chemical signal into an electrical signal. The prepared Pt@ApoF/Ti_3_C_2_-based electrochemical NaNO_2_ biosensors demonstrate a wide detection range of 0.001–9 mM with a low detection limit of 0.425 μM. Additionally, the biosensors possess high selectivity and sensitivity while maintaining a relatively stable electrochemical sensing performance within 7 days, enabling the monitoring of NaNO_2_ in complex environments. The successful preparation of the Pt@ApoF/Ti_3_C_2_ nanohybrid materials provides a new approach for constructing efficient electrochemical biosensors, offering a simple and rapid method for detecting NaNO_2_ in complex environments.

## 1. Introduction

Nitrites are commonly found in the human living environment and are among the most prevalent nitrogen-containing compounds in nature. Bearing a resemblance to table salt in appearance, nitrites are extensively utilized in industry and construction and are permitted in small quantities in certain meat products. However, excessive intake of nitrites can lead to poisoning and even death, while their long-term consumption can increase the risk of cancer [1,2,3]. Moreover, the presence of excessive nitrites in industrial wastewater can pollute water bodies, accumulate in aquatic organisms, and ultimately pose harm to humans [4,5]. In 2017, the World Health Organization classified nitrites as Group 2A carcinogens, prompting many countries to enact laws and regulations regarding their use. While these measures have mitigated the harm of nitrites to the environment and organisms to some extent, there remains a lack of a comprehensive detection and control system for nitrites [6]. Therefore, establishing a complete nitrite monitoring system is crucial for reducing nitrite poisoning and fatalities.

Currently, in order to monitor and detect the usage and content of nitrites, various detection techniques have been established. Colorimetric and chromatographic methods have been successfully applied in nitrite detection, yielding satisfactory results [7,8,9,10]. However, these methods often struggle to simultaneously meet the demands for high detection accuracy and on-site detection. As a result, electrochemical biosensors for nitrites have emerged as a promising alternative. Electrochemical biosensors offer several advantages, including low detection costs, portability, and good selectivity, making them excellent analytical tools for nitrite detection [11,12,13,14,15]. In these sensors, the electrochemical analysis of nitrites primarily involves the oxidation of nitrite ions to form nitrate products under the action of electrode materials, resulting in the generation of current signals. The linear relationship between the nitrite concentration and current signal strength is enhanced with the increase in the nitrite concentration [16,17]. The electron transfer rate of electrode materials significantly influences the oxidation rate of nitrites. Therefore, the development of novel nanomaterials with higher electron transfer rates holds great promise for advancing nitrite electrochemical biosensing technology [18,19].

Two-dimensional nanomaterials (2DNMs) possess the largest specific surface area among their counterparts, making them highly advantageous for various applications. Moreover, their facile functionalization renders them excellent substrates for synthesizing electrode materials [20,21]. Two-dimensional nanomaterials effectively optimize the concentration of active species and enhance the sensing efficacy within a confined electrode space. Importantly, certain 2DNMs demonstrate efficient electron transfer rates, further enhancing the sensing performance of hybrid constructs and resulting in a synergistic effect [22,23]. Among the wide array of 2DNMs, MXene materials have attracted considerable attention. In comparison to the initially discovered graphene and its derivatives, MXene materials offer more pathways for ionic movement, accelerating electron mobility and exhibiting better conductivity for electrochemical biosensors [24,25,26]. For instance, Murugan et al. synthesized hybrid-phase titanium carbide (Ti-C-T_X_) MXene nanosheets for the simultaneous detection of vital biomolecules like ascorbic acid (AA), dopamine (DA), and uric acid (UA) [27]. The experimental results underscored the exceptional electrocatalytic activity of Ti-C-T_X_-modified glassy carbon electrodes (GCE), showcasing remarkable sensitivity and stability and enabling the concurrent detection of AA, DA, and UA across the physiological pH spectrum. In another study, Li and co-workers integrated MXene with a photosensitive dye to create homogeneous photoelectrochemical sensors for sensitive phosphorus analysis [28]. Their results exhibited a significantly enhanced current intensity and stability in photoelectrochemical detection, alongside lowered detection thresholds, thus achieving robust homogeneous phosphorus determination with superior performance. These compelling findings illustrate that MXene holds great promise as an excellent substrate material for constructing electrochemical biosensors.

The utilization of ferritin as a template for loading nanoparticles onto MXene surfaces holds significant promise in advancing electrochemical biosensors. Ferritin, a protein with a pivotal role in organisms, possesses the unique ability to self-assemble into nanocages with exceptional stability and uniformity (12 nm). Its amphiphilic nature renders ferritin highly adaptable in interacting with ligands and the environment [29]. Based on this property, ferritin can be easily prepared as a core–shell-structured material for use. Compared to individual nanoparticles, core–shell structures have several advantages in biosensors. For instance, the shell can effectively enhance material’s stability and biocompatibility, while the core material can be endowed with properties such as electrocatalysis, optics, and electromagnetics. As a biomimetic protein, ferritin demonstrates excellent biocompatibility and biodegradability. Moreover, its consistent size is pivotal for producing nanoparticles of a uniform size, offering potential advantages in applications like drug delivery and bioimaging [30,31]. For instance, Muzykantov et al. engineered human ferritin light chains for targeted drug delivery to the lungs [32]. Biodistribution studies using various probes have revealed selective uptake in the lungs, showcasing ferritin’s potential in imaging or treating inflammatory lung diseases. Additionally, Jing and colleagues developed a cobalt nanoenzyme based on ferritin for visualizing clinical hepatocellular carcinoma tissue [33]. The cobalt nanoenzyme was biosynthesized within the protein shell of ferritin, enabling the specific targeting of hepatocellular carcinoma cells. Thus, ferritin serves as an exceptional platform for synthesizing nanoparticles of a controllable size and with targeted modifications.

**Scheme 1:**
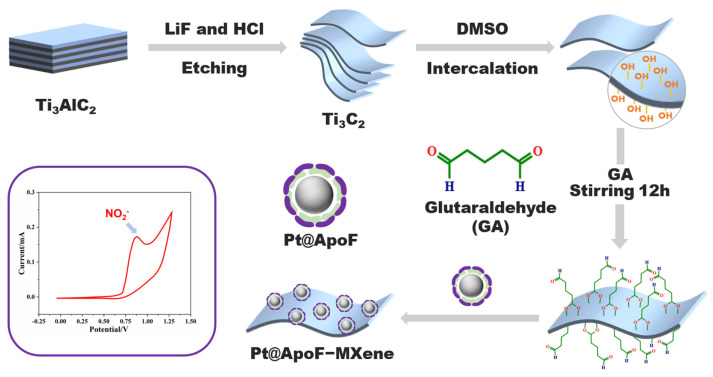
Schematic fabrication of electrochemical nitrite biosensors based on biomimetic Pt@ApoF/Ti_3_C_2_.

In this study, we developed a nitrite electrochemical biosensor based on Ti_3_C_2_ MXene materials, showcasing outstanding selectivity and sensitivity in nitrite detection. Capitalizing on the facile modification characteristic of 2DNMs, we achieved the bio-modification of MXene materials with platinum@apoferritin (Pt@ApoF) through glutaraldehyde-mediated bioconjugation, as depicted in Scheme 1. The Pt@ApoF was generated by exploiting the dissociation of ferritin in acidic or alkaline environments, followed by the removal of the iron core and modification with a platinum core through iron chelators. This method, involving the self-assembly synthesis of nanoparticles with a well-defined core–shell structure, utilizes natural cage-like proteins as templates, obviating the need for specific nucleation sites and resulting in a swifter and more convenient approach. The Pt@ApoF-modified MXene materials exhibited exceptional selectivity in the electrochemical sensing of nitrite ions, facilitating specific detection amidst numerous interfering ions. Furthermore, they demonstrated remarkable stability, maintaining consistent electrochemical performance over a testing period of up to seven days.

## 2. Materials and Methods

### 2.1. Synthesis of Ti_3_C_2_ Nanosheets

According to methods of previous reports [34,35], the etching of Ti_3_AlC_2_ with hydrofluoric acid (Shanghai Yinen Chemical Technology Co., Ltd., Shanghai, China) and the dimethyl sulfoxide (DMSO, Shanghai McLean Biochemistry Co., Ltd., Shanghai, China) intercalation of Ti_3_C_2_ nanosheets were carried out to obtain Ti_3_C_2_ monolayered nanosheets. Briefly, 1 mg of lithium fluoride (LiF, Shanghai Yinen Chemical Technology Co., Ltd., Shanghai, China) was dissolved in 20 mL of HCl (12 M, Sinopharm Chemical Reagent Co., Shanghai, China) and magnetically stirred for 10 min. Subsequently, 1 mg of Ti_3_AlC_2_ was added under stirring for 24 h in a 35 °C water bath. After that, the product was washed with 1 M HCl to remove unreacted LiF and other impurities and then washed with deionized water until the pH of the solution reached 6.0–7.0. The solution was ultrasonicated for 30 min to obtain monolayer Ti_3_C_2_ nanosheets, and the middle portion of the supernatant was collected for morphological characterizations. The clay-like multilayer Ti_3_C_2_ at the bottom of the centrifuge tube was collected and added to DMSO and reacted overnight under magnetic stirring at room temperature. The solution was dialyzed using a sodium ascorbate solution as an exchange solvent for 24 h to remove the DMSO. Finally, the monolayer Ti_3_C_2_ was obtained by freeze-drying and stored at 4 °C for subsequent experiments.

### 2.2. Synthesis of Apoferritin (ApoF)

ApoF was prepared according to a previously reported method [36]. Briefly, a horse spleen ferritin sample (Sigma-Aldrich, Saint Louis, MO, USA) was diluted 1000-fold and dialyzed in 250 mL of sodium ascorbate (NaAc, Shanghai McLean Biochemistry Co., Ltd., Shanghai, China) solution (0.1 M) at pH 5.5 for 2 h. Then, 20 μL of thioglycolic acid (TGA, Shanghai McLean Biochemistry Co., Ltd., Shanghai, China) was added. This process was repeated until the protein solution changed from yellow to colorless, resulting in the formation of ApoF. Finally, the preliminary ApoF sample was purified by dialysis in 250 mL of NaAc solution (0.1 M) at pH 5.5 for 20 min to remove the TGA. The purified ApoF solution was collected and stored at 4 °C for further use.

### 2.3. Synthesis of Platinum@Apoferritin (Pt@ApoF)

The pH of the ApoF solution was adjusted to 8 to dissociate the protein cage, and the solution was incubated at 30 °C for 30 min in the dark. Afterwards, an equal volume of K_2_PtCl_6_ (Shanghai McLean Biochemistry Co., Ltd., Shanghai, China) solution (10 mM) was added to it to stabilize it for 1 h. At the end of the reaction, a 0.1% sodium borohydride solution (20:1, *v*/*v*) was added for 30 min, followed by centrifugation (30 min, 8000 r) (Hunan Kaida Scientific Instrument Co., Ltd., Changsha, China, KH20A). After the centrifugation, the supernatant was removed, and the precipitate was redispersed in deionized water to obtain Pt@ApoF at 4 °C for further use.

### 2.4. Synthesis of Pt@ApoF/Ti_3_C_2_ Nanohybrids

**Figure 1:**
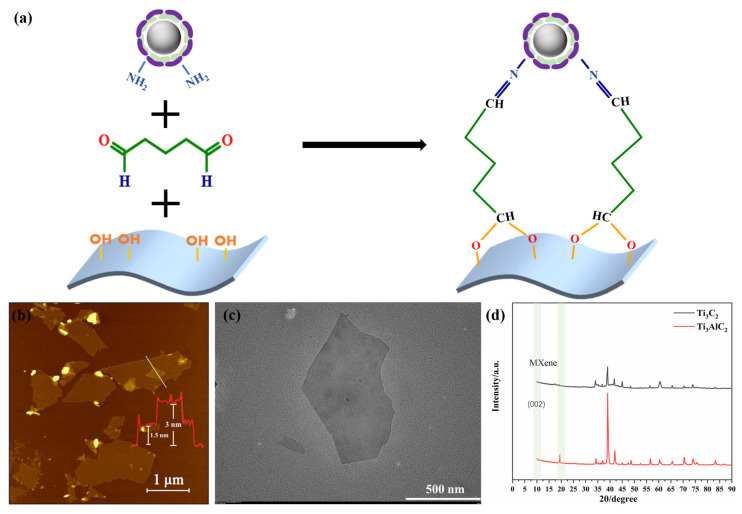
(**a**) Schematic of GA-crosslinked Pt@ApoF and Ti_3_C_2_; (**b**) AFM image of Ti_3_C_2_ nanosheets, with the number and color line in red corresponding to the monolayer and bilayer thickness of the samples; (**c**) TEM image of Ti_3_C_2_ nanosheets; (**d**) XRD image of Ti_3_C_2_ and Ti_3_AlC_2_.

The Pt@ApoF solution was mixed with the same volume of Ti_3_C_2_ solution, and then 0.1% of glutaraldehyde (GA, Sinopharm Chemical Reagent Co., Shanghai, China) at a volume ratio of 1:10 with 5 mL of Ti_3_C_2_ solution was added and stirred overnight. The unbound GA was removed by centrifugation to obtain the Pt@ApoF/Ti_3_C_2_ nanohybrid material. Through the cross-linking effect of GA, the aldehyde group at one end bound to the hydroxyl group of the Ti_3_C_2_ MXene nanosheets, while the aldehyde group at the other end bound to the amino group on the surface of the Pt@ApoF. Figure 1a shows the bonds that can be formed at the sensing interface by cross-linking the hydroxyl groups on the Ti_3_C_2_ nanosheets with the primary amino groups on the surface of the Pt@ApoF.

### 2.5. Electrochemical Detection of NaNO_2_

GCEs with a diameter of 4.0 mm were initially polished using 0.3 and 0.05 mm alumina powder, respectively, which were then ultrasonically cleaned (Dongguan Jiekang Ultrasonic equipment Co., Ltd., Dongguan, China, TUC-32) in a solution of ultrapure water and ethanol (Shanghai Testing Laboratory Equipment Co., Ltd., Shanghai, China) for 30 s (ultrasonic frequency is 40 kHz). The materials for modifying the GCEs were prepared by mixing 50 µL of Nafion solution with 1 mL of Ti_3_C_2_, Pt@ApoF, and Pt@ApoF/Ti_3_C_2_ solutions, respectively. Then, 10 μL of the modification solution was added dropwise onto the surface of the GCE to obtain the Ti_3_C_2_/GCE, Pt@ApoF/GCE, and Pt@ApoF/Ti_3_C_2_/GCE for the subsequent electrochemical tests. The samples were stored in a refrigerator at 4 °C.

### 2.6. Characterization Techniques

For the preparation of samples for atomic force microscopy (AFM) characterization, 10 μL of sample solution was deposited onto freshly cleaved mica and air-dried for the test. The AFM tests were conducted in the tapping mode in air using an FM-Nanoview 6800 AFM device (FSM-Precision, Suzhou FSM Precision Instrument Co., Ltd., Suzhou, China) at room temperature. Tap300Al-G (300 kHz, 40 N m^−1^) silicon probes (Suzhou FSM Precision Instrument Co., Ltd., Suzhou, China) were utilized for capturing the AFM images (scan image is 10,000 nm, the scanning rate is 1.01 Hz). The cantilever was calibrated when the AFM test was used for the first time every day. The height images were recorded and morphologically analyzed with the Gwyddion software (Version 2.57). The structure and morphology of the Ti_3_C_2_ and Pt@ApoF/Ti_3_C_2_ were observed by a transmission electron microscope (TEM, Tecnai G2 F20, FEI Co.; Tokyo, Japan). The samples were loaded on a copper grid and stained with phosphotungstic acid, and then the excess stain was washed off with distilled water and allowed to dry naturally. The TEM tests were performed under a vacuum microenvironment. X-ray diffraction (XRD, Smart Lab 3 kW, Rigak, Tokyo, Japan) was applied for characterizing the crystalline structure of the materials. The maximum output power of XRD was ≥3 kW, the rated voltage was ≥60 kV, and the rated current was ≥60 mA. The X-ray tube section had the following parameters: focal spot size: ≤0.4 × 12 mm; current–voltage stability: better than 0.001% (when the external voltage fluctuated by 10%); and linear range: 700,000 cps or more. The XRD using a one-dimensional detector had an effective detector area of ≥256 mm^2^. X-ray spectroscopy (XPS) was conducted with a PHI 5000 VersaProbe III spectrometer (UlVAC-PHI Company, Tokyo, Japan). The XPS instrument had an energy resolution of 0.5 eV, a spatial resolution of 50 um, a full-spectrum flux of 150 eV and a narrow-spectrum flux of 50 eV, and a full-spectrum step of 1 eV and a narrow-spectrum step of 0.1 eV. An electrochemical workstation (CHI660E, Shanghai Chenhua, Shanghai, China) was utilized for the electrochemical test of various GCEs using a traditional three-electrode system at room temperature. The working, auxiliary, and reference electrodes were modified GCE, platinum wire, and saturated calomel electrode, respectively (C-V test: −0.1 V for init. E and low E, 1.2 V for high E and final E, and 0.05 V/s for scan rate; I-T test: init. E of 0.67 V, sample interval of 0.2, run time of 4000 s, and sensitivity of 10^−4^).

## 3. Results and Discussion

### 3.1. Characterizations of Ti_3_C_2_ and Pt@ApoF

We first characterized the prepared Ti_3_C_2_ nanosheets using the AFM technique. As depicted in Figure 1b, it was found that the Ti_3_C_2_ nanosheets were successfully synthesized through the HF acid etching and subsequent DMSO intercalation. The section analysis of the AFM images revealed a uniform height distribution of the Ti_3_C_2_ nanosheets, with the measured height of the single-layer Ti_3_C_2_ nanosheets being approximately 1.5 nm, which agrees well with the height of Ti_3_C_2_ nanosheets previously obtained in the literature [37]. Additionally, as shown in the AFM images, the brightness of the nanosheet layers was consistent, indicating that the prepared MXene sheets were highly uniform. The corresponding TEM image in Figure 1c clearly exhibits the layered structure of the Ti_3_C_2_ nanosheets, ranging in size from 200 nm to 1 μm, which was in agreement with the observations from the AFM analysis.

Figure 1d illustrates the XRD pattern of the MXene Ti_3_C_2_, where the disappearance of the 20 degree peak and the appearance of the 002 peak at 10 degrees indicate the successful etching of Al from the Ti_3_AlC_2_. Therefore, we suggest that the AFM, TEM, and XRD data collectively demonstrate that the method of etching Ti_3_AlC_2_ with HF and intercalating with DMSO successfully produced uniformly sized, well-shaped, single-layer Ti_3_C_2_ nanosheets. Furthermore, the experiments conducted in a sodium ascorbate environment mitigated the oxidation rate of the Ti_3_C_2_, reducing the likelihood of its oxidation to TiO_2_ in the air and extending the shelf life of the Ti_3_C_2_.

**Figure 2:**
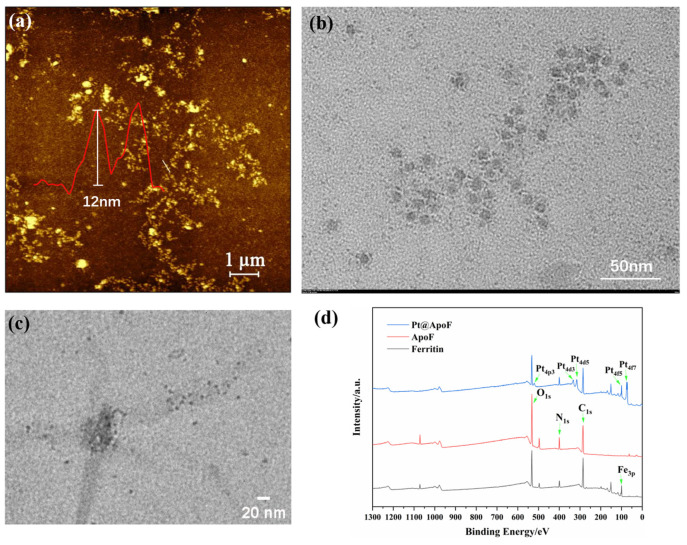
(**a**) AFM image of ferritin; (**b**) TEM image of ApoF; (**c**) TEM image of Pt@ApoF; (**d**) XPS image of ferritin, ApoF, and Pt@ApoF.

Through the obtained AFM images, the size of the ferritin was observed to be around 12 nm, consistent with the reported size of ferritin in the literature (Figure 2a) [38]. In a strong acid environment, the ferritin dissociated, and the internal iron core was removed, forming a protein nanocage with cavities, termed ApoF, as shown in Figure 2b. Adjusting the pH of the ApoF solution to 8 led to the dissociation of the protein cage. By reducing the K_2_PtCl_6_, the platinum cores were encapsulated within the cavities of the ApoF, resulting in the formation of Pt@ApoF. Figure 2c displays the TEM image of the Pt@ApoF. The XPS spectrum presented in Figure 2d clearly shows the peaks of Fe, C, N, O, and Pt. Compared with ferritin, the Fe_3p_ peak in the XPS spectrum of the ApoF noticeably disappeared, indicating the successful removal of the iron core. The XPS spectrum of the Pt@ApoF distinctly shows the characteristic peaks of Pt. Through both the TEM and XPS analysis, it was concluded that uniformly sized Pt@ApoF was successfully synthesized through biomimetic mineralization.

### 3.2. Characterizations of Pt@ApoF/Ti_3_C_2_ Nanohybrids

Ferritin-based nanomaterials are known for their remarkable stability and outstanding biocompatibility. This cross-linking reaction of GA facilitated the fabrication of the Pt@ApoF/Ti_3_C_2_ 2DNMs. Utilizing natural cage proteins as templates circumvents the issue of the nucleation sites and prevents the excessive aggregation of formed metal particles. Therefore, the biological cross-linking of Pt@ApoF with Ti_3_C_2_ enhanced the biocompatibility of the hybrid materials.

**Figure 3:**
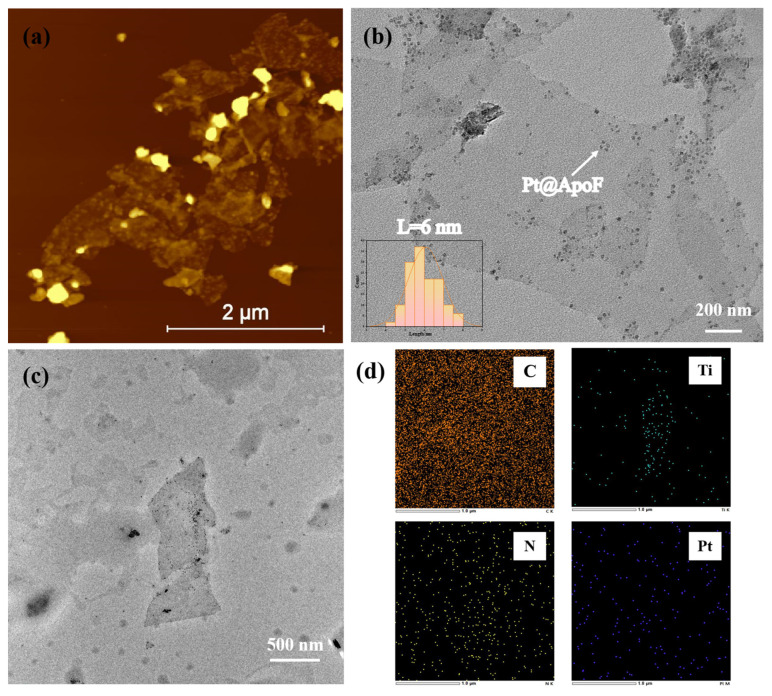
Characterizations of Pt@ApoF/Ti_3_C_2_ nanohybrids: (**a**) AFM image; (**b**) TEM image; (**c**,**d**) TEM-based elemental mapping.

Figure 3a presents the AFM image of the Pt@ApoF/Ti_3_C_2_ nanohybrid material. It is evident that numerous nanoparticles adhered to the Ti_3_C_2_ nanosheets. In Figure 3b, it can be seen that the Pt@ApoF/Ti_3_C_2_ nanohybrid material revealed uniformly distributed Pt@ApoF nanoparticles with consistent sizes on the Ti_3_C_2_ nanosheets. Additionally, further analysis indicates that the majority of the prepared Pt@ApoF particles had a diameter of about 6 nm. The corresponding TEM image and elemental spectra in Figure 3c,d were utilized for the elemental analysis of the Pt@ApoF/Ti_3_C_2_ nanohybrid material. Notably, through the mapping analysis, besides Ti and C elements from the Ti_3_C_2_, the nanohybrid material also contained N and Pt elements from the Pt@ApoF. This provides compelling evidence supporting the successful synthesis of the Pt@ApoF/Ti_3_C_2_ nanohybrid material.

**Figure 4:**
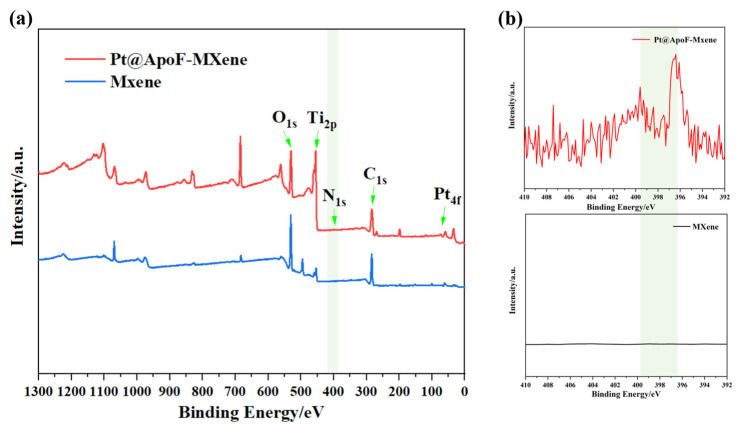
(**a**) XPS spectra of Ti_3_C_2_ and Pt@ApoF/Ti_3_C_2_ nanohybrids; (**b**) XPS spectra of N in Ti_3_C_2_ and Pt@ApoF/Ti_3_C_2_ nanohybrids (N in Pt@ApoF/Ti_3_C_2_ nanohybrids).

To better confirm the synthesis of the Ti_3_C_2_ and the formation of the Pt@ApoF/Ti_3_C_2_ nanohybrids, XPS analysis was conducted on the synthesized products. As depicted in Figure 4a, the XPS spectrum of the Pt@ApoF/Ti_3_C_2_ nanohybrid material distinctly revealed peaks corresponding to O1s, Ti2p, N1s, C1s, and Pt4f. However, the XPS spectrum of the prepared single-layer Ti_3_C_2_ nanosheets notably lacked peaks for N1s and Pt4f, further validating the successful synthesis of the Pt@ApoF/Ti_3_C_2_ nanohybrid material. Additionally, Figure 4b presents detailed XPS spectra of the Ti_3_C_2_ nanosheets and Pt@ApoF/Ti_3_C_2_ nanohybrid material, indicating that the spectrum curve of the Ti_3_C_2_ was flat without any characteristic peaks. In contrast, the spectrum of the Pt@ApoF/Ti_3_C_2_ nanohybrid material displayed a characteristic peak at 398 eV, consistent with the typical N1s peak.

### 3.3. Pt@ApoF/Ti_3_C_2_-Nanohybrid-Based Electrochemical Detection of NaNO_2_

**Figure 5:**
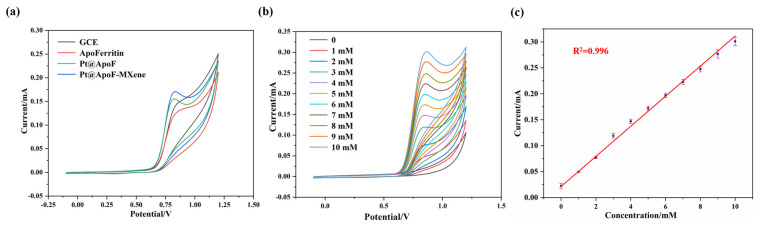
Electrochemical tests of Pt@ApoF/Ti_3_C_2_/GCE in NaNO_2_ detection: (**a**) CV curves of GCE, apoferritin/GCE, Pt@ApoF/GCE, and Pt@ApoF/Ti_3_C_2_/GCE; (**b**) current response of Pt@ApoF /Ti_3_C_2_/GCE toward NaNO_2_ with different concentrations of 0–10 mM; (**c**) linear calibration of CV response with NaNO_2_ concentration ranging from 0 to 10 mM.

In a 0.1 M NaOH electrolyte (Shanghai Testing Laboratory Equipment Co., Ltd., Shanghai, China), electrochemical tests for NaNO_2_ sensing were conducted on the synthesized Pt@ApoF/Ti_3_C_2_ nanohybrid material. Three different materials, namely, ApoF, Pt@ApoF, and Pt@ApoF/Ti_3_C_2_, were modified onto the surfaces of the GCEs to prepare functional electrodes for the CV testing. The experimental results from the CV testing reveal that the prepared Pt@ApoF/Ti_3_C_2_ nanohybrid material exhibited the strongest current signal among the three materials, with a potential range of 0.7–0.9 V (Figure 5a). MXene is a 2DNM with a high electron transfer rate and excellent conductivity, and therefore the addition of Ti_3_C_2_ nanosheets to this hybrid material system provides efficient electron transfer rates, which is favorable for the decomposition of NaNO_2_ and the generation of a corresponding current response. Furthermore, the Ti_3_C_2_ nanosheets offer a high specific surface area and abundant surface functional groups, providing rich active sites for the loading of Pt@ApoF to enhance its electrocatalytic performance.

Furthermore, CV tests were conducted for different concentrations (0–10 mM) of NaNO_2_ to further investigate the electrochemical performance of the Pt@ApoF/Ti_3_C_2_ nanohybrid material. As depicted in Figure 5b, the current response gradually increased with the increase in the NaNO_2_ concentration. The 0–10 mM NaNO_2_ concentration range exhibited an excellent linear fitting relationship, with a linear correlation coefficient R^2^ = 0.996 (Figure 5c).

**Figure 6:**
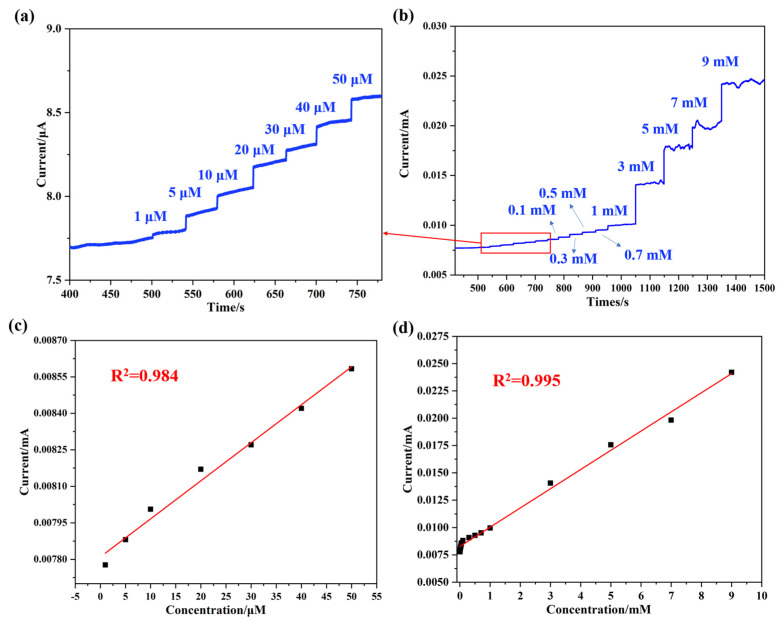
Electrochemical tests of Pt@ApoF/Ti_3_C_2_/GCE in NaNO_2_ detection: (**a**) I–T response of 0–50 μm concentrations of NaNO_2_; (**b**) I–T response of different concentrations of NaNO_2_; (**c**) linear calibration of I–T response with NaNO_2_ concentration ranging from 0–50 μm; (**d**) linear calibration of I–T response with NaNO_2_ concentration ranging from 0–9 mM.

To further explore the detection limit of the synthesized NaNO_2_ electrochemical biosensor, an I-T test was performed with the Pt@ApoF/Ti_3_C_2_/GCE. The current response peak observed at 0.8 V, as obtained from the CV curves in Figure 5a,b, was chosen as the potential for the I-T test. Figure 6a illustrates the continuous current response upon the successive addition of NaNO_2_ within the concentration range of 0–50 μM. A significant current signal was generated when the NaNO_2_ concentration in the solution reached 1 μM, demonstrating good linearity with a linear correlation coefficient of R^2^ = 0.984 (Figure 6b). It is noteworthy that as the concentration of NaNO_2_ increased, different intensity current signals were generated (Figure 6c), and there was an excellent linear fitting relationship between the current and the NaNO_2_ concentration, with a linear correlation coefficient of R^2^ = 0.995 (Figure 6d). Within the linear detection range of 0–9 mM, the calculated lowest detection limit of the Pt@ApoF/Ti_3_C_2_/GCE-based electrochemical biosensor for NaNO_2_ was 0.425 μM.

Additionally, we think that NO^2−^ ions are adsorbed onto the surface of the Pt electrode. The adsorbed nitrite ions undergo electron transfer reactions on the Pt electrode surface. In the case of oxidation, the Pt atoms on the Pt surface may provide active sites, promoting the oxidation reaction of nitrite ions. During this process, nitrite ions donate electrons to the Pt electrode, resulting in the generation of nitrate (Equations (1) and (2)).

(1) $${NO}_{2}^{-}-e^{-}\overset{K}{\to }{NO}_{2}$$

(2) $$2{NO}_{2}+H_{2}O\to {2H}^{+}+{NO}_{2}^{-}+{NO}_{3}^{-}$$

### 3.4. Selectivity and Stability of Pt@ApoF/Ti_3_C_2_ Electrochemical Platform

During the detection process of actual samples, besides NaNO_2_, other substances are often present in the samples. Therefore, electrochemical biosensors based on nanohybrid materials must exhibit excellent selectivity to sensitively detect NaNO_2_ in the presence of numerous interfering substances. Hence, selective tests were conducted on the Pt@ApoF/Ti_3_C_2_-based electrochemical biosensors.

**Figure 7:**
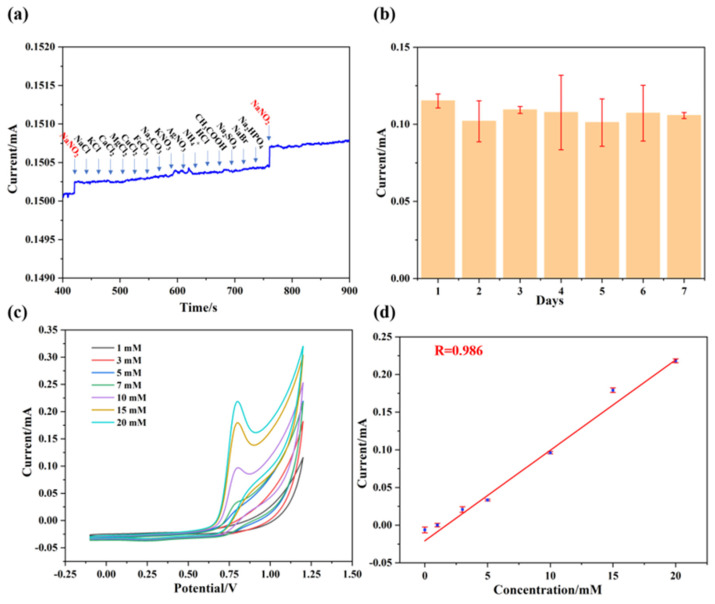
Anti-interference ability and stability of Pt@ApoF/Ti_3_C_2_−based electrochemical platform. (**a**) Amperometric responses upon successive additions of NaNO_2_, NaCl, KCl, CaCl_2_, MgCl_2_, CuCl_2_, FeCl_3_, Na_2_CO_3_, KNO_3_, AgNO_3_, NH_4_^+^, HCl, CH_3_COOH, Na_2_SO_4_, NaBr, Na_2_PHO_4_, and NaNO_2_; (**b**) stability of Pt@ApoF/Ti_3_C_2_ electrochemical platform for 7 days; (**c**) current response of Pt@ApoF/Ti_3_C_2_/GCE toward NaNO_2_ with different concentrations of 0–20 mM in milk; (**d**) linear calibration of CV response with NaNO_2_ concentration ranging from 0 to 20 mM in milk.

As depicted in Figure 7a, when NaNO_2_ was initially added, the I-T curve exhibited a significant current intensity. Subsequently, NaCl, KCl, CaCl_2_, MgCl_2_, CuCl_2_, FeCl_3_, Na_2_CO_3_, KNO_3_, AgNO_3_, NH^4+^, HCl, CH_3_COOH, Na_2_SO_4_, NaBr, and Na_2_PHO_4_ (purchased from Shanghai McLean Biochemistry Co., Ltd. (Shanghai, China)) were successively added, but no significant current response was observed in the I-T curves. However, upon the addition of NaNO_2_ again, the I-T curve once again displayed a significant current response. Therefore, we suggest that the Pt@ApoF/Ti_3_C_2_-based electrochemical biosensor exhibits excellent anti-interference capability. On the other hand, a stability test was also conducted on the hybrid nanomaterial. The Pt@ApoF/Ti_3_C_2_/GCE was placed in a −4 °C environment for 7 days, and electrochemical tests were performed daily to assess the detection ability of the Pt@ApoF/Ti_3_C_2_/GCE towards NaNO_2_. The experimental results show that the Pt@ApoF/Ti_3_C_2_/GCE exhibited consistent electrochemical performance over the 7 days (Figure 7b), indicating the good stability of the hybrid material, which maintained a high detection capability towards NaNO_2_ even after long-term storage.

Furthermore, electrochemical tests were conducted on the synthesized hybrid material using actual samples. NaNO_2_ was added to a milk sample to prepare NaNO_2_ milk solutions with different concentrations (0–20 mM) to simulate actual samples. As illustrated in Figure 7c, within the voltage range of 0.6–0.8 V, the current response increased with the increase in the NaNO_2_ content in the milk. As shown in Figure 7d, it can be observed that the electrochemical biosensor based on Pt@ApoF/Ti_3_C_2_ exhibited a good linear relationship with the actual samples. This indicates that the Pt@ApoF/Ti_3_C_2_ electrochemical biosensor maintains admirable electrochemical detection performance in actual samples and can serve as a reliable electrochemical detection platform for determining NaNO_2_.

As shown in Table 1, compared with other NaNO_2_ biosensors reported in the literature [39,40,41,42,43,44,45,46], the Pt@ApoF/Ti_3_C_2_-based electrochemical biosensor fabricated in this work has a wider detection range (0–9 mM) and a lower detection limit (0.425 μM). Based on the national regulations for the nitrite content in food, i.e., in salt, ≤ 2 mg/kg, and in fresh meat, fresh fish, grains (rice), ≤ 3 mg/kg, the minimum detection limit of NO_2_^−^ by the Pt@ApoF/Ti_3_C_2_ electrochemical biosensor was calculated to be 20 μg/kg, which is much lower than the food standards. Compared to traditional electrochemical biosensors, the preparation of the Pt@ApoF/Ti_3_C_2_-based electrochemical biosensor is environmentally friendly and without pollution, and there is no additional waste generated during the detection process. In addition, the use of ferritin as a template avoids the operation of preparing nucleation sites while increasing the biocompatibility. It has more advantages in applications such as the biological field and environmental detection.

## 4. Conclusions

In summary, we successfully synthesized a hybrid 2DNM based on Pt@ApoF for the electrochemical biosensing of NaNO_2_. While ferritin finds wide applications in drug delivery, recombinant vaccines, and contrast agents, this study represents the first application of ferritin in synthesizing biomimetic nanomaterials for electrochemical biosensors targeting nitrite detection. Through self-assembly, we achieved the synthesis of core–shell-structured Pt@ApoF and then loaded Pt@ApoF onto Ti_3_C_2_ nanosheets via bioconjugation, resulting in the successful preparation of Pt@ApoF/Ti_3_C_2_. The experimental findings demonstrate that the Pt@ApoF/Ti_3_C_2_-nanohybrid-material-based electrochemical biosensor exhibited good stability, high sensitivity, and excellent selectivity in detecting NaNO_2_. By designing the synthesis of protein–nanomaterial hybrids, we enhanced the biocompatibility of the hybrids, thus paving the way for promising applications of bioengineered 2DNMs in biomedicine, environmental science, and other fields.

## Figures and Tables

**Table 1:** Comparison of the sensing performances of various electrochemical NaNO_2_ sensors.

Materials	Linear Range [mM]	Limit of Detection [μM]	Ref.
Pt@ApoF/Ti_3_C_2_	0.001–9	0.425	This work
MnO_2_/PANI/GCE	0.1–10	4.38	[39]
MoO_3_/Co_3_O_4_/CG	0.1–1	0.013	[40]
TiO_2_-Ti_3_C_2_T_X_/CTAB/CS/GCE	0.003–0.25, 0.25–1.25	0.85	[41]
Ag/Cu/MWCNTs/GCE	0.001–1	0.2	[42]
HOOC-MWCNT/GCE	0.1–0.7	0.565	[43]
Cu^2+^-Cu^+^/Biochar/GCE	0.001–0.3	0.2	[44]
Cu/MWCNTs/GCE	0.005–1.26	1.8	[45]
Poly(TazoCoPc)/CNP/GCE	0.00002–0.001	0.006	[46]

## Data Availability

Data can be obtained by request to the authors.
